# Outcomes of Heart Failure Admissions Under Observation Versus Short Inpatient Stay

**DOI:** 10.1161/JAHA.117.007944

**Published:** 2018-01-31

**Authors:** Ahmad Masri, Andrew D. Althouse, Jeffrey McKibben, Floyd Thoma, Michael Mathier, Ravi Ramani, Jeffrey Teuteberg, Oscar Marroquin, Joon S. Lee, Suresh R. Mulukutla

**Affiliations:** ^1^ Heart and Vascular Institute University of Pittsburgh Medical Center Pittsburgh PA; ^2^ Department of Clinical Analytics University of Pittsburgh Medical Center Health Services Division Pittsburgh PA; ^3^ Division of Cardiovascular Medicine Department of Medicine Stanford University Stanford CA; ^4^ Division of Cardiology Veteran Affairs Pittsburgh Healthcare System Pittsburgh PA

**Keywords:** admission under observation, death, health policy, heart failure, outcomes research, readmission, Heart Failure, Health Services, Mortality/Survival, Ethics and Policy

## Abstract

**Background:**

Patients with heart failure (HF) are admitted either under observation (OBS) or inpatient stays; however, there is little data on whether this designation reflects the clinical status of a patient, with significant logistical and financial implications. We sought to compare the outcomes of patients with HF admitted OBS versus inpatient stay (≤2 days; INPT).

**Methods and Results:**

From January 1, 2008 to September 30, 2015, our multisite health system saw 21 339 unique patients totaling 52 493 hospital admissions with a primary diagnosis of HF. Patients were excluded if they underwent cardiac surgery (n=611), heart transplantation (n=187), or left ventricular assist device insertion (n=198), or if they died during hospitalization (n=1839). Of the remaining 50 654 discharges, 2 groups were identified: INPT group and OBS group. Outcomes were HF readmission, all‐cause readmission, and all‐cause mortality within 1 year of discharge. Hazard ratios were computed using the Andersen‐Gill method in the Cox proportional‐hazards model. A total of 8709 admissions (17%) occurred in the INPT group and 2648 admissions (5%) occurred in the OBS group. HF readmission rate at 1 year was 55.3% in INPT versus 66.5% in OBS (hazard ratio, 0.75; 95% confidence interval, 0.71–0.80; *P*<0.01). All‐cause readmission rate at 1 year was 70.7% in INPT versus 82.5% in OBS (hazard ratio, 0.74; 95% confidence interval, 0.70–0.78; *P*<0.01). All‐cause mortality at 1 year occurred in 25.2% of INPT versus 24.2% of OBS (hazard ratio, 1.03; 95% confidence interval, 0.95–1.12; *P*=0.46).

**Conclusions:**

HF admissions designated INPTs were associated with lower readmission rates and equivalent mortality to those designated OBS.

**Clinical Perspective**

**What Is New?**

- Despite the significant logistical and financial implications, heart failure admissions under observation were associated with a higher readmission rate compared with heart failure admission under inpatient stay (duration of stay, ≤2 days).

**What are the Clinical Implications?**

- There is a need for a treatment‐based patient‐centered approach to heart failure admissions, rather than the current time‐based admission rules.

## Introduction

It is estimated that 5.7 million Americans have heart failure (HF), and they account for >1 million admissions every year for treatment of HF in the United States.^1^ The current Center for Medicare and Medicaid Services (CMS) median 30‐day risk‐standardized readmission rate is 21.9% (interquartile range, 21%–23%) for 2015.^2^ Most such admissions are for decongestion using intravenous diuretics.^3^ Patient's response to the available therapies varies, which is one of the reasons that explain the wide variation in the length of stay (LOS) and the cost of each individual hospitalization.

Currently, patients with HF hospitalized in a short‐term care facility can be admitted either under observation status (OBS; considered an extension to outpatient settings) or inpatient stay. In 2013, CMS enforced the “2 midnights” rule, which meant that a patient had to stay at least 2 consecutive midnights at the hospital to count as an inpatient admission; otherwise, it would be counted as an admission OBS.^4^ This had significant implications on both hospitals and patients. From the perspective of an admitting physician, they have to predict upfront the response of a patient with HF admitted for decongestion to diuretic therapy, which, in turn, would be the driving reason for LOS and thus qualification for inpatient admission. From patients’ perspective, they are seeking in‐hospital treatment and likely do not realize that if they are placed in the OBS group, similar coverage rules do not apply and the whole “admission” might be treated as outpatient treatment in terms of copay.

However, the fitness of such a model and its interaction with the characteristics and outcomes of patients with HF admitted OBS versus short inpatient stay (<2 midnights; INPT) is yet to be defined. Patients admitted OBS status should be the least sick of all patients encountered in short‐term care settings. However, the outcomes of this group were not studied before. Thus, we hypothesized that patients who are admitted OBS have either similar or even better outcomes compared with patients admitted under short inpatient stay.

## Methods

The data, analytic methods, and study materials will not be made available to other researchers for purposes of reproducing the results or replicating the procedure.

The present study was an observational cohort study that evaluated patients admitted with a primary diagnosis of HF from January 1, 2008 through September 30, 2015, at a large multisite single healthcare system (18 hospitals). For this study, both admissions designated OBS and under inpatient status were considered as “admissions” and counted towards the total number of readmissions. Data were collected as part of an institutional HF quality improvement project, and the study was preapproved by the Quality Improvement Review Committee. The requirement for individual informed consent was waived.

Admission with a primary HF diagnosis was established through the *International Classification of Diseases, Ninth Revision* (*ICD‐9*), during that period, irrespective of left ventricular ejection fraction (LVEF). All clinical data were collected from electronic healthcare records. Various comorbidities and procedures were noted through *ICD‐9*, Current Procedural Terminology, and Healthcare Common Procedure Coding System codes and billing data (Table S1). *ICD‐10* codes were used for follow‐up visits after its institution. Patients were excluded if they underwent any cardiac surgery, cardiac transplantation, and ventricular assist device implantation or if they died during the admission. Patients were classified into groups on the basis of the admission type and the hospital LOS. The final cohort consisted of 2 groups. The first group consisted of patients admitted under inpatient designation and who stayed in the hospital for a short period (defined as <2 midnights; INPT group). The second group consisted of patients admitted to the hospital OBS. Admission type (OBS versus INPT) was based on the final admission status of the patient. The patient population studied was an all‐payer population.

### Outcomes

Four main outcomes were defined and reported at 1, 3, and 12 months. HF readmission was defined as any subsequent admission for a primary diagnosis of HF. Cardiac readmission was defined as any subsequent admission for various causes that are related to the heart. All‐cause readmission was defined as any admission irrespective of the underlying cause. All‐cause mortality was derived from the social security death index (obtained from the updated Social Security Administration Death Master file, where our healthcare system is certified by the Social Security Administration as an organization that is exempt from the 3‐year delay) and on review of the death certificate or expiration summary. To account for the individual differences among patients, we present multivariable analyses adjusted for variables that differed significantly between INPT and OBS groups (model 1: age, sex, LVEF, hypertension, diabetes mellitus, chronic obstructive pulmonary disease, pneumonia, renal failure, and liver disease). We also present multivariable analyses adjusted for these covariates plus additional clinically relevant variables (model 2: all variables in model 1 plus coronary artery disease, peripheral vascular disease, and selected cardiac medications). In a similar manner, a sensitivity analysis was performed after excluding all interventional procedures (left‐sided heart catheterization, right‐sided heart catheterization, percutaneous coronary intervention, internal cardioverter‐defibrillator implantation, and permanent pacemaker implantation).

### Statistical Analysis

Descriptive characteristics are presented as mean±SD for continuous variables or frequency (percentage) for categorical variables. This study was designed to evaluate the outcome of every patient encounter (either OBS or INPT) rather than the outcomes of the individual patient during the follow‐up of the study. Thus, every patient encounter was considered as an index admission, with every subsequent admission after that index encounter considered as a readmission. Although there are differences between the 2 groups, as reflected by the respective *P* values, every admission was regarded as an additional data point, meaning that every patient could be represented multiple times in INPT and OBS groups; thus, these reported characteristics are not entirely “independent” observations. Appropriate testing methods are used to account for multiple appearances per patient. Kaplan‐Meier estimates are presented to show the proportion of patients reaching each end point after live discharge from each HF hospitalization. Hazard ratios (HRs) for patients admitted under inpatient status versus observation status are computed using the Andersen‐Gill method.^5^ This method is an extension of the Cox proportional‐hazards model with a counting‐process structure that allows for appropriate modeling of subjects who appear more than once in a data set by imposing correlation on the repeated appearances from the same subject. Multivariable regression models were used to determine whether the HRs were affected by potential confounding variables. Statistical analyses were performed using SAS, version 9.4 (SAS Institute, Cary, NC).

## Results

**Figure 1:**
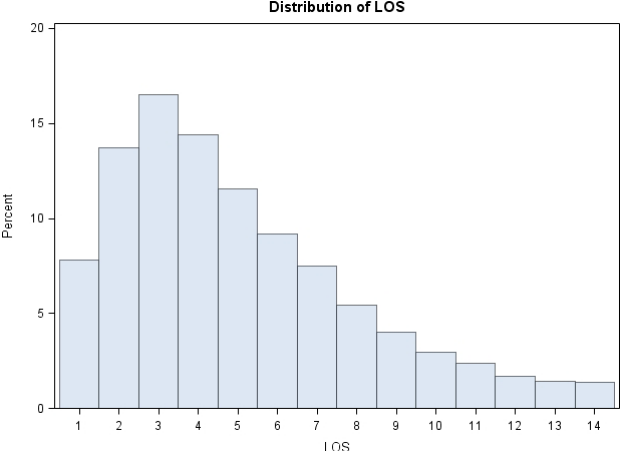
Histogram of length of stay (LOS) for the overall study population (live discharges without left ventricular assist device insertion, heart transplantation, or major cardiac surgical procedures).

During the study period, we identified a total of 21 339 unique patients with a total of 52 493 hospital admissions with a primary diagnosis of HF. We excluded 611 patients who underwent various cardiac surgical interventions, 187 patients who underwent heart transplantation, 198 patients who underwent ventricular assist device implantation, and 1839 patients who died during hospitalization. This strategy yielded 50 654 live discharges. The distribution of the LOS during the first 14 days of admission is shown in Figure 1.

### Study Cohort

**Figure 2:**
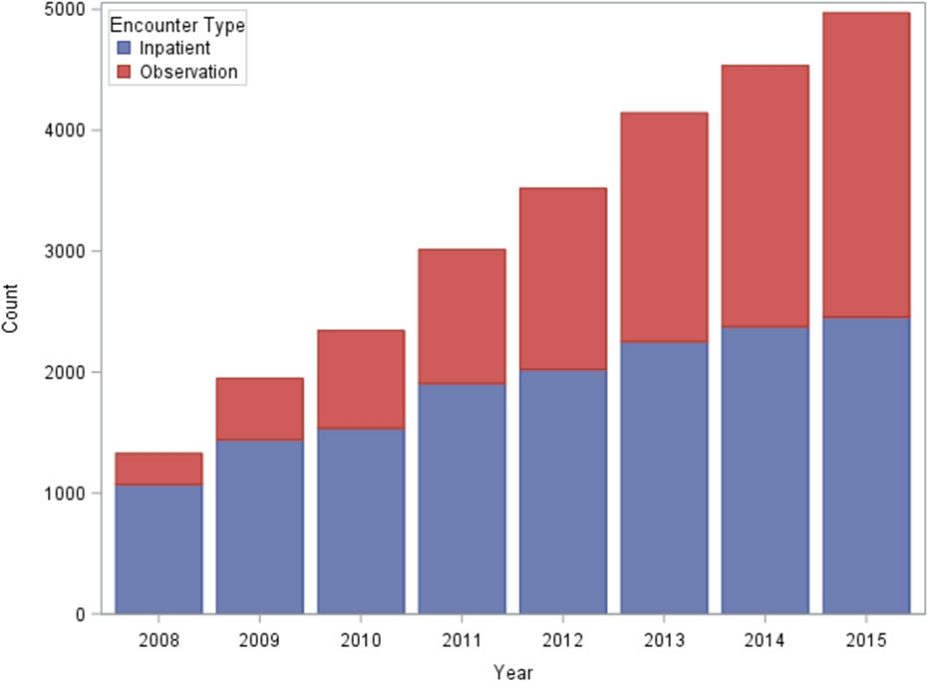
Temporal distribution of each patient's encounter under observation vs inpatient stay from January 1, 2008 through September 30, 2015.

Of the remaining 50 654 discharges, there were 8709 admissions (17%) designated as INPT and 2648 admissions (5%) designated as OBS, totaling 11 355 admissions included in the primary comparisons. All patients spent <2 days in the hospital; 38.7% of patients in OBS versus 34.8% of patients in INPT stayed 1 day. Temporal distribution of both groups over the years of the study is shown in Figure 2. Baseline characteristics, procedures, and medications of the total cohort as well as the individual groups are shown in Table 1. Mean age was 72.1±14.9 years in INPT versus 69.1±15.2 years in OBS, with a slightly higher percentage of men in INPT (51.7%) compared with OBS (47.8%). Mean LVEF in the total cohort was 41.0±16.2%, with 3612 patients (31.8%) having an LVEF of <40% (30.5% of INPT versus 36.3% of OBS had an LVEF of ≤40%). Of the total cohort, 57.5% had coronary artery disease. A high percentage of patients had multiple comorbidities, including hypertension, diabetes mellitus, peripheral vascular disease, renal disease, and liver disease (Table 1).

**Table 1:** Baseline Characteristics of the Study Cohort

Characteristics	Overall	INPT Group	OBS Group	*P* Value
No. of admissions	11 355	8707	2648	…
Descriptive characteristics				
Age, mean±SD, y	71.4±15.0	72.1±14.9	69.1±15.2	<0.001
Male sex	5766 (50.8)	4499 (51.7)	1267 (47.8)	<0.001
Hypertension	5892 (51.9)	4456 (51.2)	1436 (54.2)	0.006
Diabetes mellitus	5081 (44.7)	3798 (43.6)	1283 (48.5)	<0.001
Coronary atherosclerosis	6530 (57.5)	4994 (57.4)	1536 (58.0)	0.553
Peripheral vascular disease	1176 (10.4)	882 (10.1)	294 (11.1)	0.150
COPD	1325 (11.7)	952 (10.9)	373 (14.1)	<0.001
Pneumonia	1030 (9.1)	763 (8.8)	267 (10.1)	0.038
Renal disease	3982 (35.1)	3099 (35.6)	883 (33.3)	0.033
Liver disease	308 (2.7)	213 (2.4)	95 (3.6)	0.001
LVEF				
Mean±SD, %	41.0±16.2	40.4±16.2	41.2±16.2	0.055
LVEF <40%	3612 (31.8)	2652 (30.5)	960 (36.3)	0.023
Interventions				
LHC	209 (1.8)	172 (2.0)	37 (1.4)	0.052
RHC	51 (0.4)	32 (0.4)	19 (0.7)	0.018
PCI	111 (1.0)	108 (1.2)	3 (0.1)	<0.001
Permanent pacemaker	35 (0.3)	32 (0.4)	3 (0.1)	0.038
ICD	148 (1.3)	138 (1.6)	10 (0.4)	<0.001
Medications				
β Blocker	8404 (74.0)	1973 (74.5)	6431 (73.9)	0.925
ACE	5790 (51.0)	1495 (56.5)	4295 (49.3)	<0.001
ARB	1701 (15.0)	429 (16.2)	1272 (14.6)	0.067
Statins	7365 (64.9)	1740 (65.7)	5625 (64.6)	0.641

Data are given as number (percentage) unless otherwise indicated. ACE indicates angiotensin‐converting enzyme; ARB, angiotensin receptor blocker; COPD, chronic obstructive pulmonary disease; ICD, implantable cardioverter‐defibrillator; INPT, admitted under inpatient and discharged in ≤2 days; LHC, left‐sided heart catheterization; LVEF, left ventricular ejection fraction; OBS, under observation; PCI, percutaneous coronary intervention; and RHC, right‐sided heart catheterization.

As expected, patients admitted with HF who underwent interventional procedures (left‐sided heart catheterization, right‐sided heart catheterization, percutaneous coronary intervention, internal cardioverter‐defibrillator implantation, or permanent pacemaker implantation) were more likely to be admitted under short inpatient status (1–2 days). Only 3.5% of the entire cohort underwent such procedures.

### Outcomes

#### HF readmissions

**Figure 3:**
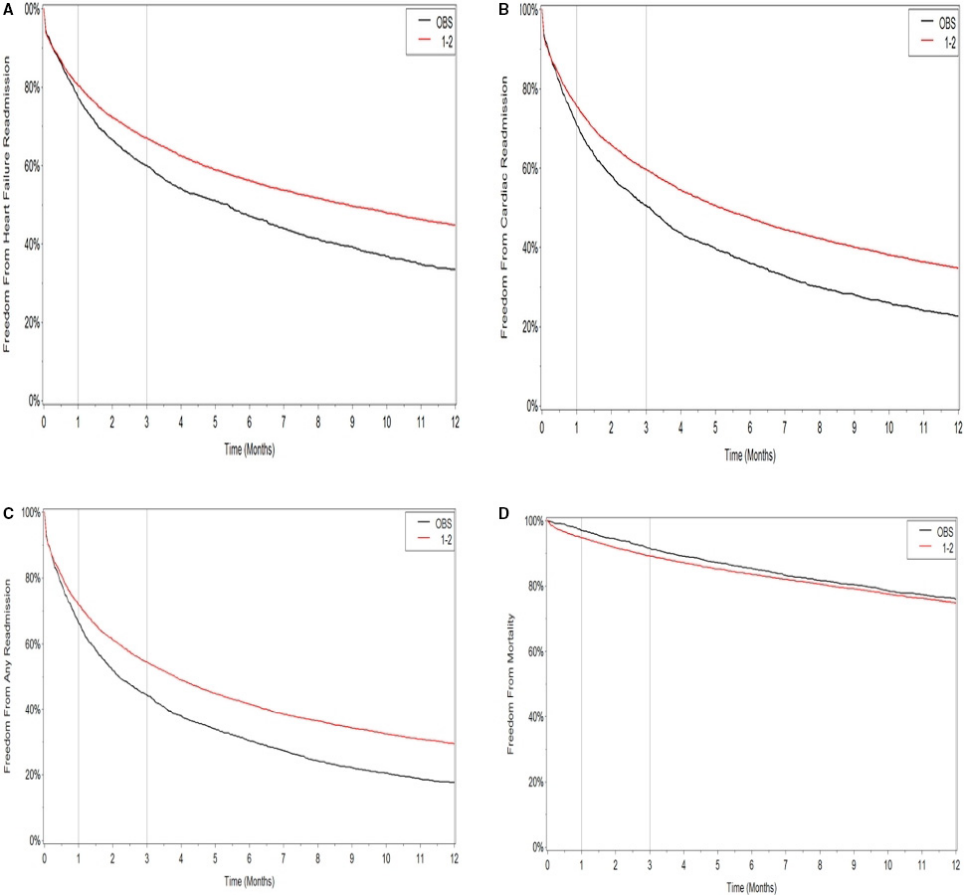
Outcomes of patients admitted under observation (OBS) vs inpatient stay and discharged in ≤2 days (1–2). A, Freedom from heart failure readmission. B, Freedom from readmission for a cardiac cause. C, Freedom from readmission for any cause. D, Freedom from mortality.

Rates of HF readmissions were lower in INPT versus OBS at 1 month (19.5% versus 22.7%), at 3 months (33.2% versus 40.3%), and at 12 months (55.3% versus 66.5%) (Table 2). Admission under inpatient status was associated with lower incidence of HF readmission compared with admission OBS (unadjusted HR, 0.75; 95% confidence interval [CI], 0.71–0.80; *P*<0.01) (Table 3, Figure 3A). This difference persisted after adjusting for baseline characteristics in model 1 (HR, 0.77; 95% CI, 0.72–0.81; *P*<0.01) and in model 2 (HR, 0.77; 95% CI, 0.73–0.82; *P*<0.01). Results were similarly consistent for the end points of cardiac readmission (Table 3, Figure 3B) and all‐cause readmission (Table 3, Figure 3C).

**Table 2:** Overall Readmission and Mortality Rates After Hospital Discharge With Primary Diagnosis of HF

Variable	INPT Group	OBS Group
CHF readmission		
1 mo	19.5	22.7
3 mo	33.2	40.3
1 y	55.3	66.5
Cardiac readmission		
1 mo	24.4	29.2
3 mo	40.6	49.8
1 y	65.3	77.4
Any readmission		
1 mo	28.1	33.6
3 mo	45.9	56.0
1 y	70.7	82.5
Death		
1 mo	5.2	3.0
3 mo	10.9	8.8
1 y	25.2	24.2

Data are given as percentage in each group. CHF indicates congestive HF; HF, heart failure; INPT, admitted under inpatient and discharged in ≤2 days; and OBS, under observation.

**Table 3:** Comparison of the Outcomes at 1 Year of Patients in the INPT Group Compared With Patients in the OBS Group (Reference Group)

Outcome	Unadjusted			Adjusted Model 1^a^			Adjusted Model 2^b^		
	HR	95% CI	*P* Value	HR	95% CI	*P* Value	HR	95% CI	*P* Value
HF readmission	0.75	0.71–0.80	<0.01	0.77	0.72–0.81	<0.01	0.77	0.73–0.82	<0.01
Cardiac readmission	0.74	0.70–0.78	<0.01	0.76	0.72–0.80	<0.01	0.76	0.72–0.80	<0.01
All‐cause readmission	0.74	0.70–0.77	<0.01	0.76	0.72–0.80	<0.01	0.76	0.72–0.80	<0.01
All‐cause mortality	1.03	0.95–1.12	0.46	0.95	0.88–1.03	0.19	0.95	0.88–1.03	0.23

On the basis of N=11 355 HF hospitalizations (8707 INPT vs 2648 OBS admissions). CI indicates confidence interval; HF, heart failure; HR, hazard ratio; INPT, admitted under inpatient and discharged in ≤2 days; and OBS, under observation.

a Model 1 adjusted for age, sex, left ventricular ejection fraction, hypertension, diabetes mellitus, chronic obstructive pulmonary disease, pneumonia, renal failure, and liver disease.

b Model 2 adjusted for effects in model 1 plus coronary atherosclerosis, peripheral vascular disease, β blockers, angiotensin‐converting enzyme, angiotensin receptor blocker, and statins.

#### Mortality

All‐cause mortality rates were numerically higher (but statistically nonsignificant) in the INPT group compared with the OBS group at 1 month (5.2% versus 3%), at 3 months (10.9% versus 8.8%), and at 12 months (25.2% versus 24.2%) (Table 2). On Cox proportional hazard analysis, there was no statistically significant different between the 2 groups, with admission under inpatient status associated with an unadjusted HR of 1.03 (95% CI, 0.95–1.12; *P*=0.46) (Table 3). Kaplan‐Meier curve is shown in Figure 3D. This persisted with adjusting for baseline characteristics in model 1 (HR, 0.95; 95% CI, 0.88–1.03; *P*=0.19) and in model 2 (HR, 0.95; 95% CI, 0.88–1.03; *P*=0.23).

Although the total number of patients who underwent interventional procedures was low, we performed a sensitivity analysis to exclude all patients who underwent an interventional procedure, and the results were identical, as presented in Table 3.

## Discussion

Our study shows that for patients with HF, OBS compared with INPT was associated with higher rates of HF, cardiac readmission, and all‐cause readmission, even after adjusting for baseline characteristics and LVEF, although the overall mortality was similar. This contradicts the assumption that patients put under observation status are low‐risk patients and should be the “least sick” of all patients with HF. Thus, the findings of this study suggest that the current admission system is a mere reflection of administrative designation, is not based on actual supportive data in patients with HF, is not reflective of actual patient status, and does not necessarily lead to an improvement in patient care; as such, the designation is somewhat arbitrary and driven primarily by insurance providers as opposed to patient‐centric measures of healthcare delivery.

The rate of HF readmission in this study was 19.5% in patients admitted under inpatient stay versus 22.7% in patients admitted OBS. Overall, this is close to the median readmission rate of 21.9% that has been reported by CMS for 2015.^2^ However, these numbers do not necessarily represent the same definition of readmission because we considered any admission (including those OBS or after an OBS admission) as a readmission. Also, we did not include all patients with HF, just those with a short hospital stay. There was not a statistically significant difference in mortality between the 2 groups, even after adjustment for baseline characteristics and LVEF. This highly argues that this system of admission OBS versus inpatient stay is arbitrary and does not necessarily reflect patient status.

OBS was founded on the notion that patients do not meet criteria for inpatient treatment but they need to be put under observed settings, and it is considered to be a continuum of the outpatient care. However, over time, this concept has transformed into a time‐sensitive definition, and patients who stay in the hospital for <2 days or 2 midnights should not be considered as inpatient admissions, with certain exceptions.^4^ Although this can be easily applied to various medical diseases for which the time course and response to therapy can be predicted, it is not practical in patients with HF and does not necessarily lead to better patient care or better healthcare delivery. Physicians and other healthcare providers are required to determine ahead of time if a patient with HF will stay >2 nights and what kind of therapies and procedures the patient might require. Because most patients with HF get admitted for decongestion^3^ and many have a variable response to therapy, affecting the LOS, it is hard to imagine that physicians can apply this in a beneficial way. On the contrary, this likely has led to more resource use from an administrative perspective.

Multiple prior studies evaluated LOS and HF readmission and mortality. For example, Reynolds et al studied LOS in 19 927 hospitalized patients with HF, and using 3‐ to 4‐day LOS as a reference group, it was shown that longer LOS was associated with worse readmission and mortality at 30 days and 1 year, whereas shorter LOS (<3–4 days) was not.^6^ Contrary to that, a post‐hoc analysis of the EVEREST (Efficacy of Vasopressin Antagonism in Heart Failure Outcome Study with Tolvaptan) study, which enrolled 4020 patients in 20 countries, has shown that a longer LOS was associated with a lower HF readmission rate at 30 days, but higher all‐cause, cardiovascular non‐HF, and noncardiovascular readmissions.^7^ Regardless of these conflicting results, the goal of this study was not to reevaluate LOS and outcomes, but to understand and evaluate the outcomes between patients admitted under short inpatient stay versus OBS. In this context, our study and its findings are unique and provide greater insight into the clinical consequences of patients with HF admitted under these different administrative designations.

Hospitalization for HF is thought to be an inflection point, where it has been shown before to be a predictor of mortality.^8^ However, this association is subject to question when evaluating all comers given the heterogeneity of the HF group, in which some present because of gaps in care and others present because of noncompliance, or having precipitant factors rather than necessarily worsening disease state. This is another important finding of this current study: even patients who are put under observation and who are not considered to be “admitted” still have a similar mortality rate to the patients who are admitted under short inpatient stay with a worse readmission rate. Given that, establishing a different admission system could help in better risk stratifying patients and help guide their prognosis and predict outcomes.

This study has multiple significant implications. First, admissions OBS do not count against the readmission rate for each hospital, and subsequent admissions after observation status do not count as readmissions. Such financial implications can lead to skewed incentives, in turn leading to practice patterns that may not be in the patients’ best interest. In an independent analysis of CMS data,^9^ it was shown that the top 10% of hospitals with the largest decrease in the rate of readmission between 2011 and 2012 had an increase in observation status use within 30 days of the initial admission by an average of 25%. This analysis was related to all readmission and not HF‐specific admissions.^9^ Second, ≈20% of patients with HF end up being discharged to a skilled nursing facility.^10^ This becomes crucial in the care of patients with HF, given under CMS rules, patients must meet the minimum 3‐day inpatient admission before placement at a skilled nursing facility.^11^ Hence, admission of a patient with HF in the OBS group can potentially make the postdischarge placement challenging. Third, insurance coverage of hospital services while patients admitted OBS versus inpatient stay varies, which, in some instances, can lead to unexpected medical bills because most patients do not recognize the different administrative designations of “being in the hospital.”^12^

Many experts have suggested a role for an established observation unit for patients with HF presenting to the emergency department. In many instances, patients just present for decongestion, and many have acute dyspnea relief in the emergency department,^13^ with some having complete resolution within 24 hours. In a viewpoint by Collins et al, the authors reviewed the current evidence and suggested a streamlined method to identify patients who benefit from observation unit placement versus high‐risk patients who need to be placed under inpatient admission.^14^ Such an approach requires a comprehensive systematic algorithm starting at the time of evaluating patients with HF in the emergency department or outpatient clinic. Our data are in support for the need for an alternative approach to replace the current system of admitting patients with HF to observation or short inpatient stay.

### Limitations

This was a retrospective observational study that evaluated patients on the basis of admission type. The aim of our study was to evaluate the outcome of every single patient's encounter OBS and short inpatient stay rather than the outcome of a cohort of patients with HF. Therefore, our database includes multiple appearances for patients in both study arms; however, we have used appropriate statistical methods to perform comparisons of the 2 groups while accounting for multiple appearances per patient. Also, the study cohort is a highly selected subgroup of patients with HF presenting to our healthcare system and, thus, the characteristics and the outcomes are not generalizable to the overall population with HF. Limiting the inclusion criteria to patients admitted OBS and short inpatient stay does, in principle, introduce selection bias. However, the study was designed to test the hypothesis that patients with HF are assigned arbitrarily to such designations. Ideally, patients with an OBS designation should be the least sick of all patients encountered in short‐term care settings. Contrary to that, our current study shows that readmission rates are lower for patients admitted under short inpatient stay, supporting the hypothesis that these designations do not reflect the patient's status at admission. Another limitation is the lack of patients’ clinical data, including vital signs, weight, and diuretic dose used. However, even if patients admitted OBS were sicker than patients admitted under inpatient stay, this still is a proof that the current admission rules are not patient centered.

## Conclusion

Many patients with HF present for decongestion without the need for additional treatment or expensive testing. However, patients are still arbitrarily assigned to be placed under either observation or short inpatient stay. In this current study, there was no mortality difference, but there was an association with higher readmission rates in patients with HF designated OBS compared with those designated INPT. This suggests that the difference between these groups is more administrative designation than a true reflection of patient status at admission. There is a need for a patient‐centered streamlined approach in evaluating and treating patients with HF with a revised treatment‐based algorithm and admission rules that guide physicians and shape healthcare policy.

## Author Contributions

Masri, Althouse, Mulukutla: designed the study, performed data extraction and analysis, and wrote and revised the article. Althouse, McKibben, Thoma: performed data extraction and statistical analysis. Mathier, Ramani, Teuteberg, Marroquin, Lee: designed the study and edited and revised the article.

## Disclosures

Teuteberg is on the advisory board of HeartWare, Abiomed, CareDx, and Acorda Therapeutics; receives speaking fees from HeartWare and CareDx; and is on the clinical events committee of St Jude's. The remaining authors have no disclosures to report.

## Supporting information


**Table S1.** ICD, CPT, and HCPCS Codes Used in Search Strategy
