# Supplementary material for: Outcomes of Heart Failure Admissions Under Observation Versus Short Inpatient Stay
Source: J Am Heart Assoc. 2018 Jan 31;7(3):e007944. doi: 10.1161/JAHA.117.007944 (PMC5850263; doi:10.1161/JAHA.117.007944)
Supplement: Supplementary file 1 — Table S1. ICD, CPT, and HCPCS Codes Used in Search Strategy [file JAH3-7-e007944-s001.pdf]

SUPPLEMENTAL MATERIAL

Table S1. ICD, CPT, and HCPCS codes used in search strategy.

CONGESTIVE HEART FAILURE	428.0,	428,	428.1,	428.20,	428.21,	428.22,
	428.23,	428.30,	428.31,	428.32,	428.33,	428.40,
	428.41,	428.42,	428.43,	428.9		
CHRONIC ISCHEMIC HEART DISEASE	414.8	414.9				
CHRONIC OBSTRUCTIVE PULMONARY DISEASE	491.xx	492	492.8	493.xx	494	494.1
CORONARY ATHEROSCLEROSIS	414.xx					
DIABETES	250.xx					
HYPERTENSION	401.0	401.1	401.9			
PERIPHERAL VASCULAR DISEASE	440.xx	441.xx	442.xx	443.xx	445.xx	
UNSTABLE ANGINA	411.1					
ACUTE CHRONIC RENAL FAILURE	484.x	485.x	586			
PNEUMONIA	480.x	482.x	483.x	484.x	485	
ACUTE CHRONIC LIVER DISEASE	571.xx	572.x	573.x	570		

HEART TRANsPLANT	37.51,	37.52,	37.53,	37.54,	33.6,	02YA0Z0,	02YA0Z1,	02YA0Z2,	02RK0JZ,	02RL0JZ,
	02WA0JZ,	0BYM0Z0,	0BYM0Z1,	0BYM0Z2						
VAD	37.60,	37.62,	37.63,	37.64,	37.65,	37.66,	37.67,	37.68,	02HA0RS,	02HA3RS,
	02HA4RS,	5A02116,	5A02216,	02HA0RZ,	02HA3RZ,	02HA4RZ,	02WA0QZ,	02WA0RZ,	02WA3QZ,	02WA3RZ,
	02WA4QZ,	02WA4RZ,	02PA0RZ,	02PA3RZ,	02PA4RZ,	02HA0QZ,	02HA3QZ,	02HA4QZ,	02QA0ZZ,	02QA3ZZ,
	02QA4ZZ,	5A0211D,	5A0221D							
CABG	36.10,	36.11,	36.12,	36.13,	36.14,	36.15,	36.16,	36.17,	36.19,	0210093,
	0210493,	02100A3,	02100J3,	02100K3,	02100Z3,	02104A3,	02104J3,	02104K3,	02104Z3,	021009W,
	02100AW,	02100JW,	02100KW,	021049W,	02104AW,	02104JW,	02104KW,	021109W,	02110AW,	02110JW,
	02110KW,	021149W,	02114AW,	02114JW,	02114KW,	021209W,	02120AW,	02120JW,	02120KW,	021249W,
	02124AW,	02124JW,	02124KW,	021309W,	02130AW,	02130JW,	02130KW,	021349W,	02134AW,	02134JW,
	02134KW,	0210098,	0210099,	0210498,	0210499,	021009C,	02100A8,	02100A9,	02100AC,	02100J8,
	02100J9,	02100JC,	02100K8,	02100K9,	02100KC,	02100Z8,	02100Z9,	02100ZC,	021049C,	02104A8,
	02104A9,	02104AC,	02104J8,	02104J9,	02104JC,	02104K8,	02104K9,	02104KC,	02104Z8,	02104Z9,
	02104ZC,	0211098,	0211099,	0211498,	0211499,	021109C,	02110A8,	02110A9,	02110AC,	02110J8,
	02110J9,	02110JC,	02110K8,	02110K9,	02110KC,	02110Z8,	02110Z9,	02110ZC,	021149C,	02114A8,
	02114A9,	02114AC,	02114J8,	02114J9,	02114JC,	02114K8,	02114K9,	02114KC,	02114Z8,	02114Z9,
	02114ZC,	021209C,	02120AC,	02120JC,	02120KC,	02120ZC,	021249C,	02124AC,	02124JC,	02124KC,
	02124ZC,	021309C,	02130AC,	02130JC,	02130KC,	02130ZC,	021349C,	02134AC,	02134JC,	02134KC,
	02134ZC,	021009F,	02100AF,	02100JF,	02100KF,	02100ZF,	021049F,	02104AF,	02104JF,	02104KF,
	02104ZF									
Valves										
TAVR	35.05,	35.06,	02RF37Z,	02RF38Z,	02RF3JZ,	02RF3KZ,	02RF37H,	02RF38H,	02RF3JH,	02RF3KH
SAVR	35.21,	35.22,	02RF07Z,	02RF08Z,	02RF0KZ,	02RF47Z,	02RF48Z,	02RF4KZ,	02RF0JZ,	02RF4JZ
MITRAL	35.23,	35.24,	02RG07Z,	02RG08Z,	02RG0KZ,	02RG37Z,	02RG38Z,	02RG3KZ,	02RG47Z,	02RG48Z
	02RG4KZ,	02RG0JZ,	02RG3JZ,	02RG4JZ						
PULMONARY	35.25,	35.26,	02RH07Z,	02RH08Z,	02RH0KZ,	02RH47Z,	02RH48Z,	02RH4KZ,	02RH0JZ,	02RH4JZ
TRICUSPID	35.27,	35.28,	02RJ07Z,	02RJ08Z,	02RJ0KZ,	02RJ47Z,	02RJ48Z,	02RJ4KZ,	02RJ0JZ,	02RJ4JZ
